# Physical and Chemical Interplay Between the Membrane and a Prototypical Potassium Channel Reconstituted on a Lipid Bilayer Platform

**DOI:** 10.3389/fnmol.2021.634121

**Published:** 2021-02-26

**Authors:** Masayuki Iwamoto, Shigetoshi Oiki

**Affiliations:** ^1^Department of Molecular Neuroscience, Faculty of Medical Sciences, University of Fukui, Fukui, Japan; ^2^Biomedical Imaging Research Center, University of Fukui, Fukui, Japan

**Keywords:** contact bubble bilayer, asymmetric membrane, bilayer tension, membrane sterol, potassium channel, KcsA

## Abstract

Once membrane potential changes or ligand binding activates the ion channel, the activity of the channel is finely modulated by the fluctuating membrane environment, involving local lipid composition and membrane tension. In the age of post-structural biology, the factors in the membrane that affect the ion channel function and how they affect it are a central concern among ion channel researchers. This review presents our strategies for elucidating the molecular mechanism of membrane effects on ion channel activity. The membrane’s diverse and intricate effects consist of chemical and physical processes. These elements can be quantified separately using lipid bilayer methods, in which a membrane is reconstructed only from the components of interest. In our advanced lipid bilayer platform (contact bubble bilayer, CBB), physical features of the membrane, such as tension, are freely controlled. We have elucidated how the specific lipid or membrane tension modulates the gating of a prototypical potassium channel, KcsA, embedded in the lipid bilayer. Our results reveal the molecular mechanism of the channel for sensing and responding to the membrane environment.

## Introduction

The chemical and physical environment of the membrane surrounding the ion channel fluctuates continuously. In living cell membranes, local lipid composition is altered by the transient formation of lipid rafts (Simons and Ikonen, 1997) or by trans-leaflet migration of lipids (flip-flop) (Higgins, 1994). Membrane tension varies by osmolality-induced cell swelling and is locally perturbed by endocytosis, exocytosis, and caveola formation (Gauthier et al., 2012; Diz-Muñoz et al., 2013). Such environmental changes in the membrane evoke modification in the ion channel’s activity via chemical and physical processes (Tillman and Cascio, 2003; Lee, 2004; Morris, 2011; Jiang and Gonen, 2012). For example, negatively charged anionic lipids or cholesterols in the membrane behave as a cofactor, supporting or modulating the K^+^ channel activity (Heginbotham et al., 1998; Huang et al., 1998; Cheng et al., 2011; Levitan et al., 2014). Moreover, membrane tension pulls the gate and opens the mechanosensitive ion channel (Martinac et al., 1990; Sachs, 2010; Douguet and Honoré, 2019). The gate regulation by such physical force from the lipid bilayer, not from the cytoskeleton or the extracellular matrix, is recognized as “force-from-lipid” gating (Anishkin et al., 2014; Cox et al., 2017). It was recently shown that membrane tension also affects the activities of channels other than so-called mechanosensitive channels, such as voltage- or pH-dependent ion channels (Morris, 2011; Iwamoto and Oiki, 2018). Thus, the “force-from-lipid” gating behavior might be a somewhat common property among ion channels. Although structural information is essential for understanding the molecular mechanism of the ion channel, the membrane-dependent feature of the ion channel function can be elucidated exclusively by functional measurements. Until recently, patch-clamp methods have been applied especially for examining mechanosensitivity. However, inherent problems such as the generation of “background” tension during gigaseal formation (Opsahl and Webb, 1994) limit further quantitative evaluation. Therefore, in the age of post-structural biology, the examination of channel-membrane interplay and the associated methodology have gained the interests of ion channel researchers.

The cell membrane consists of various lipids and membrane proteins, and it is difficult to experimentally control the membrane conditions around the channel to be analyzed. In lipid bilayer methods, a membrane is reconstructed only from the components of interest (e.g., phosphatidylcholine), and its chemical and physical features are therefore potentially controllable (Maher and Allen, 2018; Oiki and Iwamoto, 2018). Experiments using the lipid bilayer can reveal which membrane properties affect the channel activity and to what extent. This review will summarize the advanced lipid bilayer technique known as the contact bubble bilayer (CBB) method and its applications to the study of channel-membrane interplay. Using a prototypical potassium channel, KcsA, we demonstrated the anionic lipid effect and membrane tension sensitivity. Here, we describe the potential common molecular properties of the ion channel that are sensitive to the fluctuating membrane environment.

## Recent Progress in Lipid Bilayer Research

The conventional lipid bilayer (planar lipid bilayer, PLB) is formed in a small hole (diameter, approximately 100 μm) on the septum between two chambers filled with electrolytes (Latorre and Alvarez, 1981; Montal, 1987; Oiki, 2012). Ion channel molecules are then embedded in the PLB through membrane fusion. With this method, the membrane potential, lipid composition, and asymmetry between the leaflets are controllable. Consequently, the environment around the embedded channel molecules is clearly defined. These features are beneficial for the elucidation of the molecular properties of channels using electrophysiological measurements. For example, using PLB, the single-channel current properties of the TRPM8 channel were characterized by various physical and chemical stimuli (Zakharian et al., 2010). However, the PLB method requires considerable skill for the formation of a stable lipid bilayer and is regarded as a difficult technique for newcomers.

Recently, the PLB method has rapidly progressed into an easy-to-use technique. Funakoshi et al. demonstrated a novel methodology for PLB formation (Funakoshi et al., 2006). They prepared two water droplets in lipid-dispersed oil (water-in-oil droplets), allowing a lipid monolayer to form spontaneously at the water-oil interface. Then, the droplets were docked with each other to form a lipid bilayer at the contact plane. This droplet interface bilayer (DIB) method has become widespread because of extensive application to ion channel research (Malmstadt et al., 2006; Holden et al., 2007; Bayley et al., 2008; Yanagisawa et al., 2011; Urakubo et al., 2019). As the DIB method does not require any special skills, it is now becoming mainstream in lipid bilayer experiments.

**FIGURE 1:**
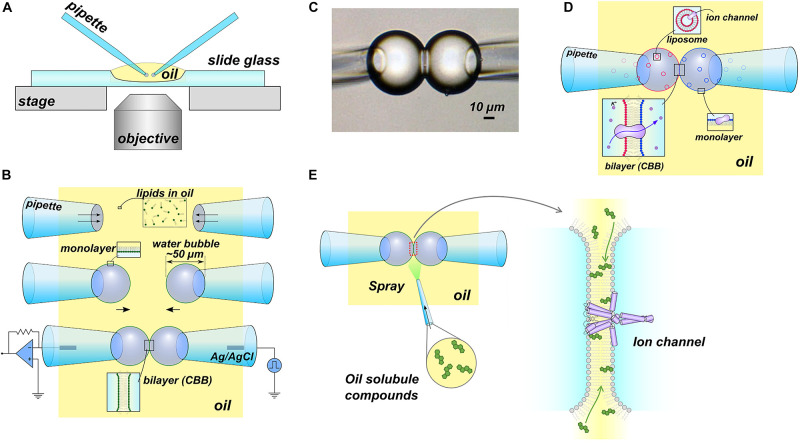
The contact bubble bilayer (CBB) experiments. **(A)** Overview of the CBB experimental setup. All experiments were performed using an inverted microscope. **(B)** Schematic illustration of the CBB formation procedure. In the simplest case, phospholipids are contained in the oil phase (e.g., 20 mg/mL azolectin in hexadecane). Two glass pipettes filled with electrolyte solutions were dipped into the lipid-containing oil (top), and a water bubble was inflated at the tip of the glass pipettes (middle). Lipid molecules in oil are spontaneously distributed at the interface between the oil and the electrolyte, forming a lipid monolayer. The two bubbles are brought into contact with each other and form a CBB (lower). **(C)** Photograph of CBB. **(D)** Schematic illustration of asymmetric CBB formation. To form an asymmetric lipid bilayer, each bubble formed with lipid-free oil (hexadecane) should contain liposomes (depicted by blue or red circles) with a different lipid composition. **(E)** Scheme for “membrane perfusion.” In the DIB methods, including the CBB method, the bilayer interior is open to the bulk oil phase. Thus, the injection of hydrophobic substances (e.g., cholesterol) in the oil phase transfers the substance to the lipid bilayer (membrane perfusion). The illustrations in **(A–D)** (Iwamoto and Oiki, 2015) and **(E)** (Iwamoto and Oiki, 2017) are modified from the original papers, respectively.

In 2015, we developed the CBB method (Figures 1A–C), which is a more versatile platform than DIB, enabling manipulation of the lipid bilayer condition, such as lipid composition and tension, during the single-channel current recording (Iwamoto and Oiki, 2015, 2018). In the CBB method, two small water bubbles (<100 μm diameter) are inflated at the tip of glass pipettes in oil and brought into contact with each other to form a small lipid bilayer (<30 μm diameter) (Iwamoto and Oiki, 2019). Hydrophobic substances, such as membrane sterols, are administered directly to the lipid bilayer via the oil phase during the single-channel current recording (Iwamoto and Oiki, 2017). An outstanding feature of the CBB method lies in the variable lipid bilayer tension through manipulation of the pressure in the bubble (Figure 1B). The pressure inside the bubble correlates with the bubble surface (lipid monolayer) tension and the lipid bilayer tension through the Young-Laplace (γ_mo_ = *R*Δ*P*/2; γ_mo_, the surface tension; *R*, the bubble radius; Δ*P*, the bubble inflating pressure) and Young (γ_bi_ = 2γ_mo_ cosθ; γ_bi,_ bilayer tension; θ, contact angle) equations (Taylor et al., 2015), respectively. Thus, the lipid composition as well as the lipid bilayer force are under control in the CBB method (Iwamoto and Oiki, 2018). This contrasts with the DIB method, where the pressure of the droplet is uncontrolled during the experiment. Furthermore, the tension operability of the CBB surpasses that of the patch-clamp method, which is a standard technique for mechanosensitive channel research. In the patch-clamp method, only the operation of relatively high membrane tension (approximately >4 mN/m) is possible (Opsahl and Webb, 1994), whereas the CBB method operates near the physiological membrane tension (<1 mN/m) (Iwamoto and Oiki, 2018). CBB experiments have advanced the understanding of channel-membrane interplay using a prototypical potassium channel as described in the following section.

## Membrane Effects on a Prototypical Ion Channel

### The KcsA Potassium Channel

The KcsA potassium channel from *Streptomyces lividans* is a pH-gated channel that opens its gate when the cytoplasmic side becomes acidic (Schrempf et al., 1995; Heginbotham et al., 1999; LeMasurier et al., 2001). The crystal structure of the KcsA channel was first resolved among ion channel proteins (Doyle et al., 1998), and its high-resolution structural information is described in detail. A noteworthy feature of the KcsA channel is that it consists only of the core structure (namely, the ion-conducting pore, gate, and ion selectivity filter) shared by various ion channels. Therefore, the KcsA channel has been regarded as a prototypical ion channel, exhibiting essential properties such as gating and selective ion conduction (Oiki, 2015). We examined the chemical and physical effects of lipid bilayers on the KcsA channel gating using the PLB and CBB methods.

### Anionic Lipid Effect

Although the KcsA channel is activated at acidic pH, its activity is significantly low in anionic lipid-free membranes (Heginbotham et al., 1998), and the negatively charged anionic lipids are thought to render the KcsA channel fully activated (the anionic lipid effect). The binding of anionic lipids to the KcsA channel was verified biochemically (Valiyaveetil et al., 2002; Marius et al., 2005), and the crystal structure showed lipid-binding at the extracellular half of the transmembrane domain (Zhou et al., 2001). Therefore, a hypothesis emerged that the binding of anionic lipids from extracellular leaflets is essential for channel activity. However, this hypothesis has remained experimentally unproven for many years.

**FIGURE 2:**
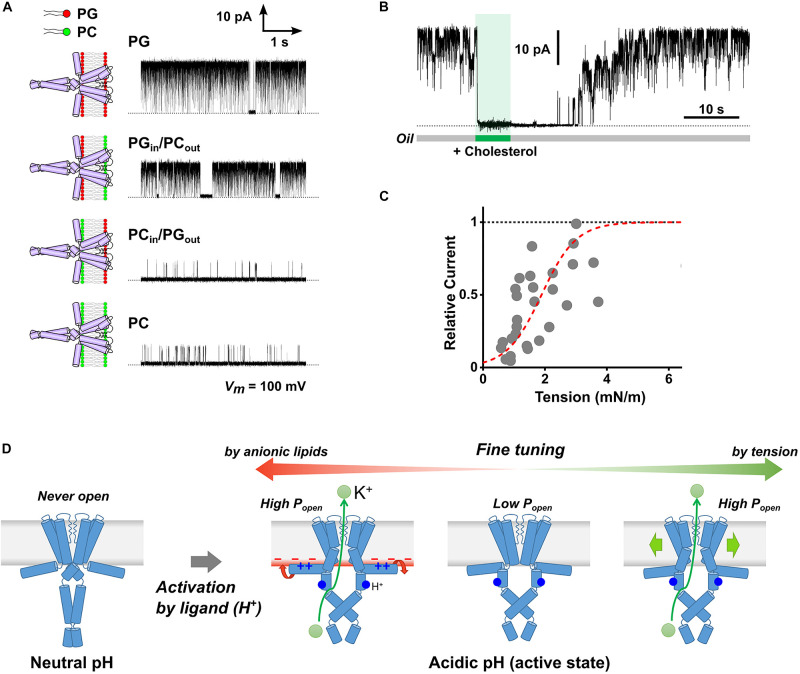
Membrane effects on the KcsA channel activity. **(A)** Typical single-channel current traces of the KcsA channel in the symmetric and asymmetric membranes under acidic pH. The membranes were formed using phosphatidylglycerol (PG, anionic) and phosphatidylcholine (PC, neutral). The electrolyte solution contained 200 mM KCl, and the membrane potential was 100 mV. **(B)** Inhibitory effect of cholesterol on the KcsA channel. The current was immediately attenuated upon cholesterol administration to the lipid bilayer (5 mg/ml) by membrane perfusion. The channel current was recovered in the original level some time after the perfusion was stopped, indicating that cholesterol diffused from the membrane; the number of channels in the membrane was constant throughout the perfusion process. **(C)** Membrane tension dependency of KcsA channel activity. The broken red line represents a Boltzmann fit where ΔG_0_/kBT is 3.37 ± 0.37, ΔA is 7.33 ± 1.23 nm^2^, and T_1/2_ of 1.89 ± 0.14 nM/m. **(D)** Scheme for multimodal regulation of KcsA channel activity by channel-membrane interplay. When the channel remained at rest at neutral pH, it did not open even in the presence of anionic lipids or under high membrane tension. Once activated by H^+^-binding (acidic pH), the channel exhibits variable activity depending on the cytoplasmic leaflet’s lipid composition or the membrane tension. Panels **(A)** (Iwamoto and Oiki, 2015), **(B)** (Iwamoto and Oiki, 2017), and **(C)** (Iwamoto and Oiki, 2018) are modified from the original papers, respectively.

We examined the molecular mechanism of the anionic lipid effect using asymmetric PLB with different lipid compositions in the extracellular and cytoplasmic leaflets (Figure 1D; Iwamoto and Oiki, 2013). Here, the orientation of reconstituted channels in the asymmetric membrane is important, which is established in the following ways. The KcsA channel is activated only when the intracellular pH becomes acidic (Heginbotham et al., 1999). Thus, when the pH was set differently (e.g., pH4 and 7.5) for both sides of the lipid bilayer, the channel whose cytoplasmic domain faces the pH4 solution is selectively activated. The single-channel current of the KcsA channel was recorded in the asymmetric membranes prepared with anionic (PG, PS, PA), cationic (EPC), and electrically neutral (PC) phospholipids. The KcsA channel exhibited a high open probability only when the cytoplasmic leaflet contained anionic lipids regardless of the lipid composition of the extracellular leaflet (Figure 2A). These results indicate that anionic lipids affect the activity of the KcsA channel from the cytoplasmic side. Thus, our results contrasted with the long-standing hypothesis that extracellular anionic lipids are essential for high activity.

The anionic lipid-sensing sites were explored using mutant channels; the positively charged amino acid residues were neutralized one at a time. The mutant channels with neutralized Arg11 or Lys14 in the N-terminal helix (M0 helix) exhibited low open probability even in the anionic lipid membrane (Iwamoto and Oiki, 2013). Thus, these two amino acid residues were identified as anionic lipid-sensors. In light of these results, the question then becomes: how do these sensors on the M0 helix affect gating? The M0 helix is not a transmembrane helix, but rather lies on the cytoplasmic membrane surface because of its amphipathicity (Perozo et al., 1998). We analyzed the lipid-sensing mechanism through the M0 helix as follows. Hydrophobic environment-sensitive fluorescence dye was attached to the site of interest of the M0 helix one at a time. Then, the fluorescence change was measured to evaluate changes in the configuration of the M0 helix upon activation. The results indicated that the M0 helix rolled around the helix axis on the membrane surface upon activation of the channel. Helix rotation did not occur without anionic lipids in the membrane. Accordingly, the anionic-lipid-dependent rotation of the M0 helix is transmitted to the gate and stabilizes the open-gate conformation of the channel (the “roll-and-stabilize” model, Figure 2D; Iwamoto and Oiki, 2013). The amphipathic helix corresponding to the M0 helix of KcsA exists in the most closely related Kir channels (Kuo et al., 2003; Whorton and MacKinnon, 2013) as well as in voltage-gated channels (Long et al., 2007; Payandeh et al., 2011). Therefore, it is expected that various channels sense the lipid composition of the membrane via the amphipathic helix, thus fine-tuning their activity.

### Membrane Tension Effect

Various mechanosensitive channels, which open the gate depending on membrane tension, have been discovered (Guharay and Sachs, 1984; Martinac et al., 1990; Coste et al., 2010; Brohawn et al., 2014; Ranade et al., 2015; Cox et al., 2016; Jojoa-Cruz et al., 2018). While the pH-gated KcsA channel usually stays deactivated with the closed gate under neutral pH, Martinac’s group examined whether the membrane tension opens the gate (Syeda et al., 2016). They set an osmotic pressure difference between two water-in-oil droplets using the DIB method to generate tension in the channel-embedded membrane. In contrast to the mechanosensitive Piezo1 and MscS channels, the KcsA channel remained closed. Consequently, they concluded that the membrane tension never activates the KcsA channel at neutral pH.

In contrast, at acidic pH, we have shown the tension-sensitive nature of the KcsA channel in the CBB experiment as follows. When cholesterol was introduced into the membrane, the KcsA channel immediately closed even at acidic pH (Figures 1E, 2B; Iwamoto and Oiki, 2017). Since cholesterol modulates the various biophysical properties of the lipid bilayer (Gimpl et al., 1997), we hypothesized that the cholesterol effect would be evoked via changes in the lipid bilayer tension or thickness. Our hypothesis was examined using single-channel current recording in the presence of various membrane sterols (cholesterol, epicholesterol, ergosterol, and lanosterol), and we obtained the following results (Iwamoto and Oiki, 2018). First, the open probability of the KcsA channel was significantly low under a high sterol concentration. Second, the membrane tension was reduced at high sterol concentrations. Consequently, the open probability was suggested to be related to the membrane tension. Further experiments were conducted using sterol-free membranes to elucidate the effect of membrane tension. In the CBB method, the surface tension of the bubble and the lipid bilayer tension are determined using the Young-Laplace and Young equations, respectively. Because the bubble inflating pressure is controllable, the lipid bilayer tension is freely manipulated in the CBB method (Taylor et al., 2015; Iwamoto and Oiki, 2018). At acidic pH, we finally showed that membrane tension higher than 2 mN/m stabilizes the open conformation of the KcsA channel (Figure 2C). Consistently with the previous report (Syeda et al., 2016), the KcsA channel never opened under neutral pH even when a higher membrane tension (>10 mN/m) was applied. These results indicate that once the KcsA channel is activated by acidic pH, the open probability is fine-tuned by relatively weak membrane tension (Figure 2D). Such a tension-dependent feature contrasts with the mechanosensitive channel, which utilizes higher membrane tension for activation (>10 mN/m for MscL) (Sukharev et al., 1999). It is possible that the activation by acidic pH can render the KcsA channel susceptible to weak membrane tension because of the changes in the flexibility of the channel molecule, which will be revealed in further studies.

## Conclusion

To date, lipid bilayer experiments have steadily advanced the understanding of channel-membrane interplay. The KcsA channel is a good research target because of its minimum structure exhibiting the essential function of the ion channel, such as gating and selective ion permeation. Using PLB and CBB, we have revealed that anionic lipids and membrane tension are required for the full activity of the KcsA channel (Figure 2D). These results provide insight into the mechanism that evokes the membrane-dependent features of the ion channel. With a broader range of tension manipulation than that of the patch-clamp method, the CBB method unveiled the response of the non-mechanosensitive channel toward weak membrane tension. We expect that the molecular mechanism for responding to weak tension adds a general aspect to the conventional mechanosensitivity, which has been studied exclusively using mechanosensitive channels. Although CBB has not yet been widely applied to the functional study of ion channels, membrane manipulability of the CBB will further advance the understanding of the “force-from-lipid” gating behavior of various ion channels. In living cells, the membrane environment around the channel fluctuates continuously, and the activity of various ion channels can be fine-tuned. Future research with the advanced lipid bilayer technique will further unveil the multimodal regulation of the ion channel and elucidate the underlying molecular mechanism regarding the utilization of the membrane environment for ion channel activity.

## Author Contributions

MI wrote the manuscript discussed with SO. Both authors contributed to the article and approved the submitted version.

## Conflict of Interest

The authors declare that the research was conducted in the absence of any commercial or financial relationships that could be construed as a potential conflict of interest.
